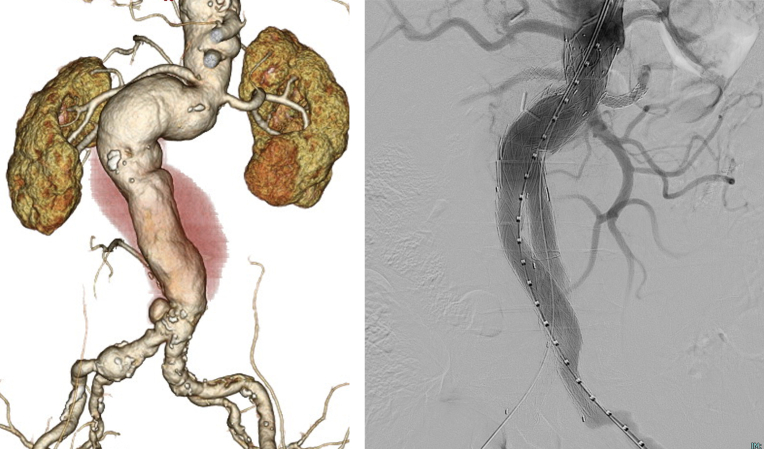# Pararenal aortic aneurysm repair using a physician-modified stent-graft with inner branches

**DOI:** 10.1016/j.jvscit.2022.04.016

**Published:** 2022-06-14

**Authors:** Tsuyoshi Shibata, Yutaka Iba, Tomohiro Nakajima, Itaru Hosaka, Nobuyoshi Kawaharada

**Affiliations:** Department of Cardiovascular Surgery, Sapporo Medical University, Sapporo, Japan

A 90-year-old man had presented with a 55-mm pararenal aortic aneurysm (expansion rate, 6 mm/mo). Gutter endoleaks have limited the use of parallel grafts. Physician-modified fenestration is one of the effective techniques in emergency cases.^1^ However, its use has posed concerns regarding the development of endoleaks from fenestration sites.^2^ In addition, company manufactured devices have a time delay. Hence, we assembled inner branched stent grafts to reduce the possibility of a type IIIc endoleak. Sapporo Medical University approved the present study (approval no. 20-005). The patient provided written informed consent for the report of his case details and imaging studies.

A sterile, 58-mm long, 26/26-mm Zenith alpha abdominal cuff (Cook Medical, Inc, Bloomington, IN) was modified. Based on the measurements from the computed tomography scan and the three-dimensional printed aortic model, an 8 × 8-mm fenestration for the superior mesenteric artery and two 6 × 6-mm fenestrations for the bilateral renal arteries were created at predetermined locations of the Dacron fabric via electrocautery. Subsequently, two inner branches were created by cutting the 6 × 6 × 100-mm Viabahn (W.L. Gore & Associates, Flagstaff, AZ) into 15-mm segments. Each inner branch was attached to the 6 × 6-mm fenestrations for the bilateral renal arteries using running Proline sutures inside the stent graft (*A*/Cover). Finally, the modified stent grafts were manually resheathed.

The stent graft system was inserted into the aorta via the left femoral artery. The fenestrations were confirmed to be in the same positions as the visceral arteries. Device deployment was fully unsheathed. A 7-mm Viabahn balloon expandable endoprosthesis (W.L. Gore & Associates) was deployed via the left brachial artery from the inner branch to the right renal artery. A similar procedure was performed for the left renal artery. Finally, the main body of the graft, an Excluder aortic stent graft (W.L. Gore & Associates), was deployed inside the modified stent grafts. Completion angiography revealed patent visceral arteries and no endoleaks (*B*). The patient recovered uneventfully.

To the best of our knowledge, three recent studies have reported on physician-modified inner branched stent grafts using an actual thoracic stent graft.^3^, ^4^, ^5^ This technique allows surgeons to achieve a longer landing zone and possibly a better seal and should be considered for urgent and emergency cases. To the best of our knowledge, our case is the first in which an aortic-modified cuff was used, which was advantageous in terms of costs and the minimum necessary extension to the sealing zone.

**Figure:**
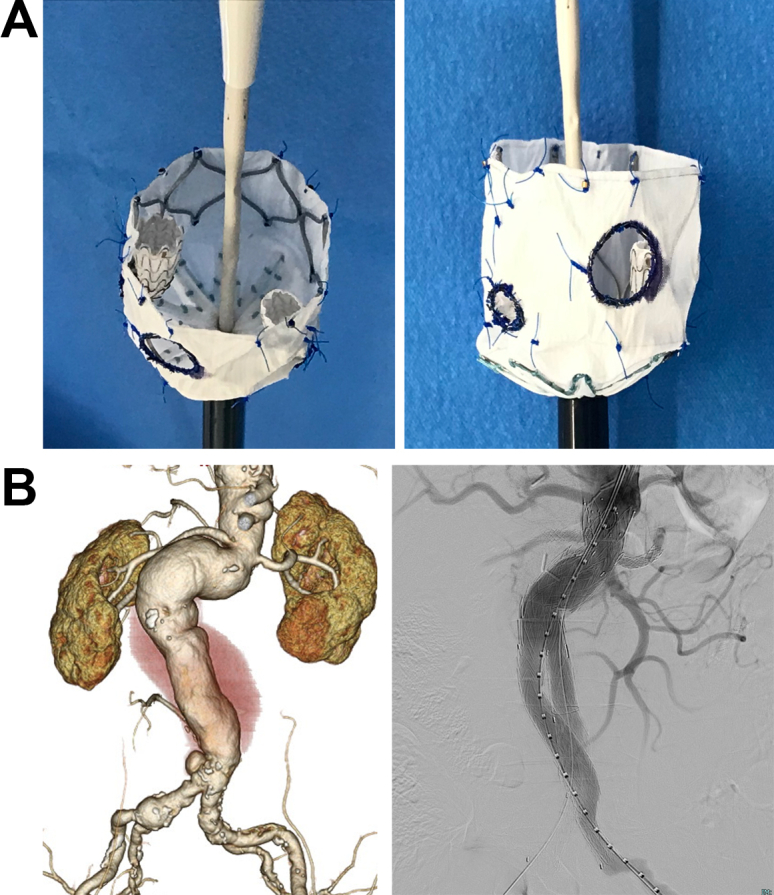

## Figures and Tables

**Cover image:**